# Updating OakEcol: A database of Oak-associated biodiversity within the UK, with data on Sita spruce associated biodiversity within the UK (Sitka spruce Ecol)

**DOI:** 10.1016/j.dib.2025.112328

**Published:** 2025-11-26

**Authors:** Ruth Joy Mitchell, Steve Derek Albon, Paul E Bellamy, Lydia Cocks, Chris J Ellis, Nick G Hodgetts, Caitlyn Johnstone, Jenni A Stockan, Andy FS Taylor

**Affiliations:** a The James Hutton Institute#,†, Craigiebuckler, Aberdeen, AB15 8QH, United Kingdom; b RSPB Centre for Conservation Science, The Lodge, Sandy, Bedfordshire, SG19 2DL, United Kingdom; c Royal Botanic Garden Edinburgh, 20A Inverleith Row, Edinburgh, EH3 5LR; d Bryophyte Consultant, Cuillin Views, 15 Earlish, Portree, Isle of Skye IV51 9XL, United Kingdom

**Keywords:** Alternative tree species, Associated species, Biodiversity, Diversification, Forestry, Resilience, Species use

## Abstract

This paper presents an update to a previously published dataset known as “OakEcol” which listed the species associated with native oak trees (*Quercus petraea/robur*) in the UK and if these species are also hosted by 30 other tree species. This new dataset “Sitka spruce Ecol” collates information on the species (birds, bryophytes, fungi, invertebrates, lichens and mammals) that are known to use Sitka spruce *Picea sitchensis* in the UK. In the UK Sitka spruce is the most commonly planted commercial tree species. The Sitka spruce Ecol database provides the first collated list of species hosted by Sitka spruce across the UK and provides the information necessary to assess if diversification of Sitka spruce plantations with other tree species will provide resilience for the Sitka spruce associated biodiversity in the event of Sitka spruce declining in abundance due to climate change or a pest or pathogen. In total 564 species are listed in Sitka spruce Ecol. For each species we provide a level of association with Sitka spruce, ranging from only found on Sitka spruce (obligate) to found on a wide range of other tree species (cosmopolitan). Information on the part of the tree used, and use made of tree (feeding, roosting, breeding) was collated for each Sitka spruce associated species. We assess if the 564 species found on Sitka spruce are also hosted by any of 34 other tree species that could be planted in mixes with Sitka spruce. Many of these 34 tree species were also assessed as alternative hosts for the oak associated biodiversity in OakEcol. The methods used to collate the data in Sitka spruce Ecol were as similar as possible to those used in OakEcol making it possible to combine the datasets for additional analysis showing the biodiversity supported when other tree species are planted with Sitka spruce.

Specifications Table

**Table:** 

Subject	Forestry
Specific subject area	Species found on Sitka spruce in the UK and if they will also use 34 other tree species.
Type of data	Raw data. Tables – CSV files with instructions on how to recreate a relational database if required.
Data collection	The approach used similar methods to those in [1,2] to collate records from the literature and databases of the birds, bryophytes, fungi, invertebrates, lichens and mammals, hosted by Sitka spruce [3]. However, the methodology differed from [1] as additional records and new literature was available from which to collate the data. The lichen data was assessed at the tree genera level rather than the tree species level [1] as the species of spruce was often not recorded. The tree species assessed as alternative hosts for Sitka spruce associated species are not the same as those tree species in OakEcol as only alternative hosts that would grow in the same environmental conditions as Sitka spruce were assessed.
Data source location	Data were collated from UK records of species occurrence.
Data accessibility	Repository name: Environmental Information Data Centre Data identification number: 10.5285/1ce52c10-e3ab-4996-b9b7–052c70b3c1ba Direct URL to data: https://doi.org/10.5285/1ce52c10-e3ab-4996-b9b7–052c70b3c1ba
Related data article	R.J., Mitchell, P.E. Bellamy, C.J. Ellis, R.L. Hewison, N.G. Hodgetts, G.R. Iason, N.A. Littlewood, S. Newey, J.A. Stockan, & A.F.S. Taylor, OakEcol: A database of Oak-associated biodiversity within the UK. *Data in Brief* 25 (2019) https://doi.org/10.1016/j.dib.2019.104120
Related research article	Ruth J Mitchell, Steve D Albon, Paul E Bellamy, Clare Cameron, Chris J Ellis, Nick G Hodgetts, Caitlyn Johnstone, Jenni A Stockan, Andy F S Taylor. Sprucing up the UK’s Sitka spruce (*Picea sitchensis*) forests: can tree species diversification benefit biodiversity?, Forestry: An International Journal of Forest Research, 2025; cpaf040, https://doi.org/10.1093/forestry/cpaf040

## 1 Value of the Data

• The most complete list of biodiversity associated with Sitka spruce, *Picea sitchensis*, in the UK.
• For each species information is provided on the level of association with Sitka spruce, ranging from obligate (only found on Sitka spruce) to cosmopolitan (found on a wide range of other tree species).
• The data can be used to assess if 34 other trees support the same biodiversity as found on Sitka spruce.
• This dataset, Sitka spruce Ecol, can be used in conjunction with databases on the Oak-associated biodiversity in the UK (OakEcol: 10.5285/22b3d41e-7c35–4c51–9e55–0f47bb845202) and Scots pine-associated biodiversity in the UK (Scots pine Ecol: 10.5285/97f8c19b-7451–4809-b011–0f9cf3eb8430) to assess how tree species diversification of commercial Sitka spruce plantations can increase the biodiversity supported.
• The dataset can be used to assess the biodiversity impacts of changes in the UK Forestry Standard requiring a diversity of tree species to be planted.

## 2 Background

This dataset (Sitka spruce Ecol) [3] follows similar methods to that used to construct the OakEcol dataset [1,2] to collate the first list across taxon groups, across the UK, of species that use Sitka spruce, termed SS-associated species. While Sitka spruce (*Picea sitchensis*) is not native to the UK, it is the UK’s most economically valuable tree species and is widely planted. Historically Sitka spruce was grown in monocultures but the UK Forestry Standard [4] is driving a change towards growing a greater diversity of tree species. To understand how the biodiversity of Sitka spruce forests may change with a move away from Sitka spruce monocultures one first needs to know what biodiversity is supported by Sitka spruce. This dataset [3] addresses this knowledge gap and provides data on whether the Sitka spruce associated species will also use 34 other tree species. This data article provides greater depth on how the data in [5] were collected and the format in which the data is held. Together with the data in [1,2,6] the data can be used, as shown by [5] to assess how tree species diversification of Sitka spruce forests may increase the biodiversity supported.

## 3 Data Description

This dataset [3] contains 11 CSV files (Table 1). The data is a list of Sitka spruce associated species (termed SS-associated species) and information about how the SS-associated species use Sitka spruce trees and if they will use 34 other tree species (Table 2). The data is text collated in a standard format, details of which are shown in Table 3, Table 4, Table 5, Table 6, Table 7, Table 8, Table 9, Table 10, Table 11, Table 12, Table 13. The structure of the files and the definitions used are as similar as possible to those used in [1,2] to enable the datasets to be combined.

**Table 1:** List of files (CSV) in the Sitka Spruce Ecol database, a description of the data they contain, and where metadata can be found.

Table (file) name	Description of data	Metadata for each file:
Species	The main data table in the database to which most other tables are related. A list of all Sitka spruce-associated species, Latin (and English names where they exist), their level of association with Sitka spruce and the confidence in the data.	Table 3
Age	The age of the Sitka spruce tree that the Sitka spruce-associated species uses.	Table 4
Alternative_tree_names	A list of 34 Latin and English tree names for which an assessment as to whether the Sitka spruce-associated species would or would not use them, was made. Includes information about the suitability of the alternative tree to grow with Stika spruce and the Forest Development Type this mixture would be related to.	Table 5
Alternative_Trees	Information on if the Sitka spruce-associated species will or will not use 34 other tree species.	Table 6
Conservation_status	Information on the conservation status of the Sitka spruce-association species	Table 7
Forest_Structure	The structure of the forest that the species uses e.g. open or closed canopy.	Table 8
Forest_Type	The forest type the species uses, e.g. ancient woodland, plantation, recent semi-natural woodland.	Table 9
References	Full details of all references used to make an assessment of the level of association of the Sitka spruce-associated species with Sitka spruce and with the alternative tree species.	Table 10
Season	The season when the Sitka spruce-associated species uses the Sitka spruce tree.	Table 11
Tree_part	The part of the tree that the Sitka spruce-associated species uses.	Table 12
Use	How the Sitka spruce-associated species uses the Sitka spruce tree e.g. feeding, roosting, nesting.	Table 13

**Table 2:** Tree species assessed for their potential to host the biodiversity supported by *Picea sitchensis*.

Latin name and authority	English name
*Abies alba* Mill.	European silver fir
*Abies amabilis* Douglas ex J.Forbes	Pacific silver fir
*Abies grandis* (Douglas ex D. Don) Lindley	Grand fir
*Abies procera* Rehder	Noble fir
*Acer pseudoplatanus* L.	Sycamore
*Alnus glutinosa* (L.) Gaertn	Common alder
*Betula pendula/pubescens* Roth	Birch (silver/downy)
*Carpinus betulus* L.	Hornbeam
*Castanea sativa* Mill.	Sweet chestnut
*Chamaecyparis lawsoniana* (A.Murray bis) Parl.	Lawson's cypress
*Cryptomeria japonica* (L.f.) D.Don	Japanese red cedar
*Cuprocyparis leylandii* A. B. Jacks. & Dallim.	Leyland cypress
*Fagus sylvatica* L.	Beech
*Larix × marschlinsii*	Hybrid larch
*Larix decidua* Mill	European larch
*Larix kaempferi* (Lamb.) Carr.	Japanese larch
*Picea abies* (L.) H. Karst.	Norway spruce
*Picea x lutzii* Little	Lutz's spruce
*Pinus contorta* Douglas	Lodgepole pine
*Pinus pinaster* Aiton	Maritime pine
*Pinus ponderosa* Douglas ex C.Lawson	Ponderosa pine
*Pinus radiata* D.Don	Radiata pine
*Pinus sylvestris* L.	Scots pine
*Populus tremula* L.	Aspen
*Prunus avium* L.	Wild cherry
*Pseudotsuga menziesii* (Mirbel) Franco	Douglas fir
*Quercus petraea/robur* (Matt.) Liebl.	Oak (sessile/pedunculate)
*Sequoia sempervirens* (D.Don) Endl.	Coast redwood
*Sequoiadendron giganteum* (Lindl.) J.Buchh	Giant redwood
*Sorbus aucuparia* L.	Rowan
*Thuja plicata* Donn ex D.Don	Western red cedar
*Tilia cordata* Mill.	Small leaved lime
*Tilia platyphyllos* Scop.	Large leaved lime
*Tsuga heterophylla* (Raf.) Sarg.	Western hemlock

**Table 3:** Details of data in Species.csv file. Definitions align where possible with those used in [1,] to enable joint use of the datasets.

Column heading name	Definition
Species_Latin	Latin name of species that uses Sitka spruce
Authority	Authority for Latin name
Synonym	Any known synonyms for the species
Species_English	English name
Group	The group of species the species belongs to: Bird, Bryophyte, Fungi, Invert-Acari Invert-Coleoptera, Invert-Diptera, Invert-Hemiptera, Invert-Hymenoptera, Invert-Insect, Invert-Lepidoptera, Invert-Other, Invert-Thysanoptera, Lichen Mammal-Artiodactyla, Mammal-bat, Mammal-carnivore, Mammal-rodent.
Group2	The group of species the species belongs to, grouped at a higher level than under group with all the invertebrates grouped together as inverts and all the mammals grouped as mammals.
Level_association	Level of association with Sitka spruce: • Obligate= Unknown from other tree species/can not complete its live cycle without Sitka spruce. • High = Rarely uses trees other than Sitka spruce • Partial = Uses Sitka spruce more frequently than its availability • Cosmopolitan = Uses Sitka spruce as frequently or lower than availability. • Uses= Uses Sitka spruce but the importance of Sitka spruce for this species is unknown. • Obligate_genera = Used when information about which trees a species uses was only held at the tree genera level, ie spruce. The species only occurs on spruce trees. • High_genera = Used when information about which trees a species uses was only held at the tree genera level, ie spruce. The species rarely uses other tree genera other than spruce. • Partial_genera = Used when information about which trees a species uses was only held at the tree genera level, ie spruce. The species uses spruce more frequently than its availability. • Cosmopolitan_genera = Used when information about which trees a species uses was only held at the tree genera level, ie spruce. The species uses spruce trees as frequently or lower than availability. • Uses_genera = Used when information about which trees a species uses was only held at the tree genera level, ie spruce. The species is known to use this tree genera but the importance of this tree genera for this species is unknown.
Association_confidence	The type of data on which the level of association is based: • Anecdotal = Information on the level of association between the species and the tree is predominantly based on anecdotal evidence. • NR-NonUK = Information on the level of association between the species and the tree is predominantly based on literature/databases that has a unknown review process and uses data from outside the UK. • NR-UK = Information on the level of association between the species and the tree is predominantly based on literature/databases that has a unknown review process but is based on UK data. • PR-NonUK = Information on the level of association between the species and the tree is predominantly based on peer reviewed literature or databases with some form of quality control but uses data from outside the UK. • PR-UK = Information on the level of association between the species and the tree is predominantly based on peer reviewed literature or databases with some form of quality control using data from the UK.
Meta-data_quality	Free text for general comments on the quality of the data used
Notes	Notes in relation to level of association with Sitka spruce
Reference_Number	Reference(s) used - look up number(s) in References table

**Table 4:** Details of data in Age.csv file.

Column heading name	Definition
Species_Latin	Latin name of species that uses Sitka spruce
Age	• Seedling/sapling = Species uses Sitka Spruce trees under 2 m in height. • Pole = Species uses the pole stage Sitka Spruce trees which are defined as >2 m height, younger than 50 years. • Mature = Species uses mature Sitka Spruce trees which are defined as >2 m in height and under 3 m in girth, don't have lots of holes, rotten wood etc. - characteristics which are veteran tree characters. • Veteran= Species uses Veteran Sitka Spruce trees which includes Veteran, Ancient and Notable trees: defined as large girth (usually over 3 m, taking into consideration environmental conditions) with at least 3 veteran attributes (e.g. important habitats visible such as dead wood in the trunk, contain standing dead wood, have fallen wood around base, rot holes, water pockets, seepage lines, hollows in trunk or major limbs etc.).
Notes	Notes to do with ecology of the species
Reference_Number	Reference used - look up number(s) in References table

**Table 5:** Details of data in Alternative_Tree_name.csv file.

Column heading name	Definition
Alternative_English	English name of alternative tree species
Alternative_Latin	Latin name of alternative tree species for which an assessment was made as to whether the Sitka spruce-associated species would or would not use this tree.
Native	Is the tree native to the UK? *Y* = Yes, *N* = No. Note that although a tree species maybe native to some countries of the UK it may not be native all countries within the UK.
Suitability	The suitability of the alternative tree to grow in intermate mixes with Sitka spruce. 1 = very suitable, 4 = less suitable. Data taken from Kerr, G., Haufe, J., Stokes, V. and Mason, B. 2020. Establishing robust species mixtures. Quarterly Journal of Forestry, 114, 164–170.
FDT	The Forest Development Type this tree species together with Sitka spruce would start to form. Data taken from Haufe, J., Kerr, G., Stokes, V. and Bathgate, S. 2021. Forest Development Types: A guide to the design an dmangement of diverse forests in Britain. Version 1.0. Forest Research.

**Table 6:** Details of data in Alternative_Trees.csv file.

Column heading name	Definition
Species_Latin	Latin name of species that uses Sitka spruce
Alternative_Latin	Latin name of alternative tree species
Association	Level of association of the Sitka spruce-associated species with that tree species: • Yes_Sp = Known to use this tree species. • Yes_Gen = Known to use this genus but no information on if it will use this species. • Rarely_Sp = The associated species has very occasionally been recorded on this tree species but very rarely, so unlikely to be a good alternative tree species. • Rarely_Gen = The associated species has very occasionally been recorded on this genus but there is no information on if it will occasionally use this species. Unlikely to be a good alternative tree species. • No = Not known to use this tree species. • Parasite = Species is a parasite, so no assessment of alternative tree species made. • Probable = Based on ecological knowledge of the species the associated species is thought likely or probable to use this tree species but there are no records of the species using this particular tree species. For example, the species is known to use a wide range of deciduous tree species, thus it is probable that it will also occur on other deciduous tree species, even if no records of its occurrence on this tree species exist. • Unknown = The use (or otherwise) of this tree is unknown.
Association_confidence	The type of data on which the level of association is based: • Anecdotal = Information on the level of association between the species and the tree is predominantly based on anecdotal evidence. • NR-NonUK = Information on the level of association between the species and the tree is predominantly based on literature/databases that has a unknown review process and uses data from outside the UK. • NR-UK = Information on the level of association between the species and the tree is predominantly based on literature/databases that has a unknown review process but is based on UK data. • PR-NonUK = Information on the level of association between the species and the tree is predominantly based on peer reviewed literature or databases with some form of quality control but uses data from outside the UK. • PR-UK = Information on the level of association between the species and the tree is predominantly based on peer reviewed literature or databases with some form of quality control using data from the UK.
Reference_Number	Reference used - look up number(s) in References table

**Table 7:** Details of data in Conservation_status.csv file.

Column heading name	Definition
Species_Latin	Latin name of species that uses Sitka spruce
Conservation_status_known	• Known_protected = Species is listed as being of some conservation concern in one of Red data, IUCN, priority species list. • Known_unprotected = Species is known to be of no conservation concern. • Unknown = The distribution/population of the species is unknown and it is not known whether this species should be of conservation concern.
Red_data_book	If the taxon group has a red data book and if the species is listed in it then the category from the red data book is listed: Critically endangered, Data deficient, Endangered, Least concern, Nationally Notable, Near threatened, Not evaluated, Vulnerable.
IUCN	If the species is listed by the IUCN then its IUCN category (Critically endangered, Endangered, Vulnerable, Near Threatened, Least concern, Data deficient) was recorded as defined by [7].
BoCC5	For Birds the Birds of Conservation Concern 5 [8] was used to categorise their conservation status as red, amber or green listed
Priority_sp_England	Indicates if the species is on the list of English Priority species. (species of principal importance) http://webarchive.nationalarchives.gov.uk/20,140,711,133,551/http://www.naturalengland.org.uk/ourwork/conservation/biodiversity/protectandmanage/habsandspeciesimportance.aspx
Priority_sp_Wales	Indicates if the species is on the list of Welsh Priority species. (Section 7 List) http://www.biodiversitywales.org.uk/Environment-Wales-Bill
Priority_sp_NI	Indicates if the species is on the list of Northern Ireland Priority species. https://www.daera-ni.gov.uk/articles/northern-ireland-priority-species
Priority_sp_Scotland	Indicates if the species is on the list of Scottish Priority species. (Scottish Biodiversity list) http://www.gov.scot/Topics/Environment/Wildlife-Habitats/16,118/Biodiversitylist/SBL

**Table 8:** Details of data in Forest_structure.csv file.

Column heading name	Definition
Species_Latin	Latin name of species that uses Sitka spruce
Forest_structure	The type of forest structure in which the species uses Sitka spruce trees. The forest structure classification was adapted from [9] and define as: • Dense low shrub layer = Low complex dense vegetation of shrubs and woody plant structures typically within 2 m of the ground. • Dense high shrub layer = Complex dense vegetation structures in the upper shrub layer typically 2–5 m above the ground. • Open understorey structure = Stands with no or little low shrub or woody vegetation (i.e. <5 m of the ground). • Open canopy structure = Woodland with significant gaps between the crowns of individual trees. Such trees may be open-grown with spreading canopies and often have relatively high amounts of dead/decaying wood. • Closed canopy - few strata = Stands where the canopy layer is relatively simple often associated with single-aged mid-growth phases. • Closed canopy - multiple strata = Stands where the canopy layer is relatively complex forming several foliage strata often associated with more mature growth phases. Multiple strata could be derived from mixtures of trees of different ages or from high canopy depth within individual trees
Notes	Notes to do with ecology of the species
Reference_Number	Reference used - look up number(s) in References table

**Table 9:** Details of data in Forest_type.csv file.

Column heading name	Definition
Species_Latin	Latin name of species that uses Sitka spruce
Forest_type	The type of forest in which the species uses Sitka spruce tree: • Ancient semi-natural woodland = any site that has always been wooded since at least 1600AD (in England and Wales) when the first maps appeared. • Recent semi-natural woodland = Woodland established since AD1600 that have regenerated and planted woodland/plantations. • Non-woodland = Trees outside woodlands such as single trees in hedges, gardens, parks or small groups of trees.
Notes	Notes to do with ecology of the species
Reference_Number	Reference used - look up number(s) in References table

**Table 10:** Details of data in References.csv file.

Column heading name	Definition
Reference_number	Unique reference number that links to the other tables in the database.
Authors	Full list of authors
Year	Year of publication
Type	Type of publication: Book, journal article, webpage, database
Paper_title	Title of paper if applicable
Publication_title	Title of publication
Volume	Journal volume
Page_numbers	Page numbers
Database_title	Title of database
Web_address	Web address if applicable
Publishers	Publishers
Editors	Full list of editors
Place_published	Place of publication
DOI	DOI

**Table 11:** Details of data in Season.csv file.

Column heading name	Definition
Species_Latin	Latin name of species that uses the Sitka spruce
Season	The season when the Sitka spruce tree is used: • Spring (March, April, May) • Summer (June, July, August) • Autumn (September, October, November) • Winter (December, January, February)
Notes	Notes to do with ecology of the species
Reference_Number	Reference used - look up number(s) in References table

**Table 12:** Details of data in Tree_part.csv file.

Column heading name	Definition
Species_Latin	Latin name of species that uses Sitka spruce
Tree_part	The part of the tree that is used. • Bark = The species grows/lives/eats/otherwise uses the bark of Sitka Spruce trees (used for lichens/bryophytes that grow on the bark of the tree and birds that hunt for insects on the bark of the tree). • Dead wood-standing = The species grows/lives/eats/otherwise uses Sitka Spruce standing dead wood. • Dead wood-fallen = The species grows/lives/eats/otherwise uses fallen dead wood from Sitka Spruce. • Dead wood-on live tree = The species grows/lives/eats/otherwise uses dead wood on live Sitka Spruce trees. • Cones = The species grows/lives/eats/otherwise uses the cones of Sitka Spruce. • Needles = The species grows/lives/eats/otherwise uses the needles of Sitka Spruce. • Limbs/branches/twigs = The species grows/lives/eats/otherwise uses the limbs/branches/twigs of Sitka Spruce. • Roots = The species grows/lives/eats/otherwise uses the roots of Sitka Spruce. • Seeds = The species grows/lives/eats/otherwise uses the seeds of Sitka Spruce. • Trunk = The species grows/lives/eats/otherwise uses the trunk of Sitka Spruce trees (this was used for birds/mammals/inverts that live/breed inside holes in trees, but bark was used for lichens/bryophytes that grow on the bark of the tree).
Notes	Notes to do with ecology of the species
Reference_Number	Reference used - look up number(s) in References table

**Table 13:** Details of data in Use.csv file.

Column heading name	Definition
Species_Latin	Latin name of species that uses Sitka spruce
Use	How the Sitka spruce-associated species uses the Sitka spruce tree: • Feeding – direct = The species eats part of the Sitka Spruce (e.g. seeds, needles, wood, bark). • Feeding – indirect = The species eats another organism found on the Sitka Spruce. • Habitat - living space = The species uses the Sitka Spruce as a habitat in which to live (e.g. epiphytes, bird nest holes etc.).
Notes	Notes to do with ecology of the species
Reference_Number	Reference used - look up number(s) in References table

The primary file is Species.csv to which most other tables are related, ie they have the same unique species names in, which could be used to make a relationship in a relational database. The Species.csv file contains a list of all SS-associated species, Latin (and English names where they exist), their level of association with Sitka spruce and the confidence in the data.

Information on the conservation status of associated species is contained in the file Conservation_status.csv (see Table 6 for associated metadata). As different taxon groups have different categories of conservation protection we broadly classified the conservation status of each associated species as to whether the species was known to have some form of conservation protection or not. More detailed information on the conservation status of the associated species was then collated using the relevant taxon specific classification such as the IUCN status of the species [7], if the species was on a UK Red Data book list, or on the priority species list of any of the four countries within the UK. The UK Birds of Conservation Concern list 5 [8] was used to assess if the bird species were of red, amber or green conservation concern.

Information on the ecology of the SS-associated species is held in six files: The Use.csv file provides data on how the species uses the tree, for feeding directly, feeding indirectly or as a living space for nesting or roosting or as a habitat for epiphytic species. Metadata and definitions for the Use.csv file are provided in Table 13. The Tree_part.csv file shows the part of the tree e.g. needles, bark, cones, used by the SS-associated species (Table 12 provides metadata and definitions). The Age.csv file shows the age of the tree used by the SS-associated species with associated metadata and definitions in Table 4. The Forest_structure.csv file indicates the structure of the forest used, whether it is an open or closed canopy and the number of strata within the forest, associated metadata and definitions are in Table 8. The Forest_type.csv file (associated metadata and definitions in Table 9) collates information on whether the SS-associated species uses ancient woods, recent woods or trees outside woodlands. The Season.csv file indicates if the SS-associated species uses the tree during the spring, summer, autumn or winter (associated metadata and definitions in Table 11).

Whether the associated species use any of the 34 alternative tree species (Table 2) is held in the file Alternative_Trees.csv (Table 6). These 34 alternative tree species are all species that have been suggested as alternative commercial tree species to Sitka spruce or species that could be used to diversify Sitka spruce plantations [5]. The assessment of use of these alternative trees was conducted in the same manner as for Sitka spruce (Table 14).

**Table 14:** Methods used to assess level of species associated with Sitka spruce. For each species the reference used is listed in the Sitka Spruce Ecol database.

Species group	Data sources and criteria used to assess association
Birds	The assessment of birds associated with *Picea sitchensis* trees was primarily based on online searches of peer reviewed literature using Web of Science and Google scholar. Search terms used where the scientific name of the Tree species + Birds initially, then for birds associated with that tree further searches for the specific bird species were used to complete all the attributes for the species. Most assessments of level of association were based on studies that directly compared bird use of several tree species within a study. This was supplemented with published observational records of tree use. Further information was sought from unpublished reviews on the habitat associations and requirements for woodland birds. See references.csv for a complete list of references. The final list of species was checked against other studies of bird communities in Sitka spruce plantations to identify if there were species likely to be using the trees that were not included elsewhere.
Bryophytes	The British Bryological Society (BBS) records https://records.nbnatlas.org and the references for individual species as listed in the references.csv file were used to assess the association of the bryophytes with Sitka spruce.
Fungi	All assessments were made based on data held in the Fungal Records Database of Britain and Ireland (FRDBI). This database is held as two databases - pre and post 2016. Data from both databases were used. In total there were 4112,000 records of which 2772,000 records had data with an associated organism. The data were cleaned by CJ and AT. Detailed assessments of fungi were limited to those with >30 % of records associated with *Picea sitchensis.* Obligate species were defined as those with 90–100 % of records with *Picea sitchensis,* a criteria of 100 % was not used as the method used to record the associated organism within the FRDBI is to record the nearest organism to the fungus, this can result in many erroneous organisms being listed. Highly associated fungi were defined as those with 50–89 % of their records associated with *Picea sitchensis.* Partially associated fungi were defined as those with 30–50 % of their records associated with *Picea sitchensis.* Due to the large number of fungi (418) which would be classified as Cosmopolitan or Uses detailed assessments for these fungi were not made. There were a number of Sitka associated records in the database of ectomycorrhizal fungi known to be highly specific on other tree species: these were removed from the final dataset.
Invertebrates	A literature review was used to collate information on the species that use *Picea sitchensis* and an assessment of their level of association was made based on the evidence within that literature. The literature used for each species is given within the database in the references.csv file.
Lichens	Data from the British Lichen Society database (1960–2022) was used to identify species associated with *Picea* sp. or spruce. All data was analysed at the genera level of tree host as the majority of host data within the database was recorded as spruce rather than individual species of spruce. Data on substrates was recorded in an inconsistent manner within the database. The data on substrates was cleaned and duplicate records removed by CJ and CE. The number of times that each species had been recorded on spruce was calculated as a proportion of the total number of all records across all substrata (including corticolous, terricolous and saxicolous records, etc.). The ‘level of association’ for a species was considered obligate if 100 % of records were from *Picea*, high if >50 % of records were from *Picea*, partial if >2.2 % of records are from *Picea*, and cosmopolitan if the number of records from *Picea* trees <2.2 %. The 2.2 % threshold was calculated by dividing the summed number of records which are from spruce trees (for those species known to occur on spruce) by the summed total number of records for the lichen species across all substratum types.For assessment of use of alternative trees: trees with fewer than 50 lichen records and lichen species with fewer than 50 records were excluded from analysis. A cluster analysis was performed to determine tree groupings for each tree species; hierarchical analysis was based on flexible beta-linkage (*b* = −0.25) applied to a Bray-Curtis coefficient matrix, and with pruning to delimit clusters at a point where the recognition of indicator species is maximised [10] implemented using PC—Ord v. 7 [11].Alternative tree categories were assigned to each lichen species based on the following rules: Lichen species must have 50 or more total records on tree species assigned to a Tree Genus. 1. Yes_Gen = at least 10 % of the records for this lichen species occur on this tree genus. 2. Probable = the tree genus occurs in a cluster from which at least 25 % of the records of the lichen species occur. 3. Probable = at least 5 % of the records for this lichen species occur on this tree genus AND the tree genus occurs in a cluster from which 20–25 % of the records of the lichen species occur. 4. Rarely = the lichen occurs on the tree AND does not meet the criteria for Yes_Gen or Probable. 5. Rarely = there is no record on this tree genus in the BLS database AND the tree genus occurs in a cluster from which 15–25 % of the records of the lichen species occur. 6. No_Gen = the lichen does not occur on the tree AND does not meet the criteria for Yes_Gen, Probable, or Rarely. 7. Unknown = the lichen has <50 total records.
Mammals	The handbook of British Mammals (Harris and Yalden, 2008). Retrieved from http://books.google.co.uk/books?id=*w*_UJNAAACAAJ was used as the main information supplemented with additional literature searches for specific species (see references.csv for details).

## 4 Experimental Design, Materials and Methods

To identify those species that use Sitka spruce (*Picea sitchensis*) in the UK (termed SS-associated species) an extensive search of both published and grey literature, together with quality controlled databases was conducted in 2023/34. The data collation was limited to six taxon groups: birds, bryophytes, invertebrates, fungi, lichens and mammals. To ensure consistency across taxon groups a pre-defined common structure was used to collate the data across taxon groups to enable the data to be used in constructing a database called Sitka spruce Ecol.

For each SS-associated species its level of association with Sitka spruce within the UK was categorised as ‘obligate’, ‘high’, ‘partial’, ‘cosmopolitan’ or ‘uses’ (Table 3). For fungi and lichens these categories were defined based on the number of records of a species occurring on/with Sitka spruce as opposed to other tree species. For birds, bryophytes, invertebrates and mammals a more subjective assessment was made based on the available literature (Table 14). A similar methodology was then used to assess if the SS-associated species did or did not use any of 34 alternative tree species (Table 5). For both the assessment of the level of association of the SS-associated species with Sitka spruce and whether the SS-associated species did or did not use the 34 alternative tree species the quality of the data used to make the assessment was recorded. This was based on whether the literature was peer reviewed/the database quality controlled and if the data were from the UK or not. In addition, all the data sources for assessments were recorded (References.csv and associated metadata and definitions in Table 10.

## Limitations

It was beyond the scope of the project to clean all the records of SS-associated fungi from the Fungal Records Database of Britain and Ireland (FRDBI). The number of SS-associated fungi is therefore an under-estimation as only obligate, high and partially associated fungi were collated. There were a further 418 SS-associated fungi identified which would be classed as cosmopolitan. In addition, the FRDBI dataset records associations with fungal fruit bodies as the nearest living organism. In mixed woodlands it is often impossible to be certain what the fungal fruit bodies are associated with. This could result in an over- or underestimation of the number of SS-associated fungi, and it is not possible to estimate the error in the data due to this.

The lichen data in the British Lichen Society database was predominately recorded at the tree genera level rather than the tree species level. Thus, the level of association record for all lichen species was at the tree genera rather than species level as the species of spruce used is unknown. However, as Sitka spruce is the most widely planted spruce species within the UK it is likely that most of these records come Sitka spruce trees.

## Ethics Statement

The authors have read and followed the ethical requirements for publication in Data in Brief and confirm that this work does not involve human subjects, animal experiments, or any data collected from social media platforms*.*

## CRediT Author Statement

**Ruth Mitchell:** Conceptualization; Methodology; Writing – Original draft. **Steve Albon:** Investigation. **Paul Bellamy:** Investigation. **Lydia Cocks:** Investigation. **Chris Ellis:** Investigation. **Nick Hodgetts:** Investigation. **Caitlyn Johnstone:** Investigation. **Jenni Stockan:** Investigation. **Andy Taylor:** Investigation.

## Acknowledgments

This work was supported by a UKRI Future of UK Treescapes grant DiversiTree: NE/X004449/1 and by the Scottish Government Rural and Environment Science and Analytical Services Strategic Research Programme 2022–2027.

## Data Availability

Environmental Information Data CentreSitka spruce-associated biodiversity in the UK (Sitka spruce Ecol) (Original data).
